# Inequalities in outcomes after kidney transplant failure in the UK

**DOI:** 10.1371/journal.pone.0356769

**Published:** 2026-08-26

**Authors:** Samuel Westaway, Pippa K. Bailey, Shalini Santhakumaran, Matthew Beresford, George Greenhall, Sian Griffin, Barnaby D. Hole, Rachel Hilton, Sherry Masoud, Dorothea Nitsch, Lucy Plumb, Matthew Robb, Maria Pippias

**Affiliations:** 1 Population Health Sciences, Bristol Medical School, Bristol, United Kingdom; 2 North Bristol NHS Trust, Bristol, United Kingdom; 3 UK Renal Registry, UK Kidney Association, Bristol, United Kingdom; 4 Gloucestershire Hospitals NHS Foundation Trust, Gloucester, United Kingdom; 5 Barts Health NHS Trust, London, United Kingdom; 6 University Hospital of Wales, Cardiff, United Kingdom; 7 Wales Kidney Research Unit, Wales, United Kingdom; 8 Guy’s and St Thomas’ NHS Foundation Trust, London, United Kingdom; 9 National Registry of Rare Kidney Diseases, Bristol, United Kingdom; 10 Centre for Kidney and Bladder Health, University College London, London, United Kingdom; 11 The London School of Hygiene and Tropical Medicine, London, United Kingdom; 12 Bristol Royal Hospital for Children, Bristol, United Kingdom; 13 NHS Blood and Transplant, Bristol, United Kingdom; Showa University, JAPAN

## Abstract

**Background:**

Failing transplant management is an international research priority. The Failing Kidney Transplant Outcomes Registry Analysis (FAKTOR) is investigating management and outcomes of kidney transplant failure in the UK.

**Methods:**

A retrospective cohort analysis using UK Renal Registry data on adults (≥18 years) with kidney transplant failure (eGFR < 15ml/min/1.73m^2^) between 01/2012-12/2021. Treatment status was determined each year post-failure as the first status recorded of: i) re-transplantation, ii) haemodialysis (HD), iii) peritoneal dialysis (PD), iv) death; those alive without changing Kidney Replacement Therapy (KRT) modality classed as v) alive without new KRT, subclassified as vi) managed conservatively if eGFR < 7.5ml/min/1.73 m^2^ with no future dialysis. Treatment status was determined by one-year post-failure and reported by: age, sex, ethnicity, socioeconomic group, primary renal disease (PRD) and centre type (transplanting/non-transplanting). When applicable, last eGFR pre-dialysis was recorded. Regression models investigated the relationship between patient characteristics and centre type with outcomes: i) treatment status by one-year and ii) pre-dialysis eGFR.

**Results:**

We included 5447 patients. By one-year, 47.8% first received HD and 5.2% were pre-emptively re-transplanted. Black and Asian people were less likely to be re-transplanted than White people (Black Relative Risk Ratio (RRR) 0.41, 95% CI 0.20–0.87. Asian RRR 0.57, 0.34–0.96). Males were more likely to be re-transplanted (RRR 1.39, 1.06–1.81) and to receive HD (RRR 1.68, 1.47–1.92). Each quintile increase in deprivation was associated with a 22% lower re-transplantation likelihood (RRR 0.78, 0.71–0.86). Diabetic PRD was associated with higher mortality (RRR 1.97, 1.34–2.88) relative to glomerulonephritis. Median pre-dialysis eGFR was 9.6 ml/min/1.73 m^2^ (IQR 7.7–11.7) and 7.1% higher in non-transplanting compared with transplanting centres (+7.1%, + 2.6% to +11.9%).

**Conclusions:**

This first nationwide description of outcomes following kidney transplant failure in the UK highlights strong evidence of pre-emptive re-transplantation inequalities, favouring White people, those in less deprived areas and males.

## Introduction

In the UK, approximately 25% of kidney transplants fail within 10 years of transplantation [1]. As the number of kidney transplants increases, the number of people with failing grafts will rise [1]. People with failing transplants experience higher rates of mortality and hospitalisation than those with native kidney failure [2]. Compared with transplant-naïve patients, haemodialysis recipients with failed transplants experience lower quality of life and biochemical measures of care quality [3,4].

Management of kidney transplant failure has been highlighted as an international research priority [5–7]. There is little high-quality evidence to guide optimal care for people with failing or failed transplants [7,8]. which may explain variation in practice. Our UK practice patterns survey demonstrated variation in the use of a failing transplant protocol, triggers for referral to a low clearance clinic, and immunosuppression modification [9,10].

The FAiling Kidney Transplant Outcomes Registry Analysis (FAKTOR) aims to investigate the management and outcomes of people with failing kidney transplants in the UK. This first output determined: i) the frequency and prevalence of different kidney treatment modalities in the five years following transplant failure, and ii) the eGFR at which people with failed transplants started dialysis. It investigated whether there was variation according to centre and patient demographics in a) the treatment status by one-year post-transplant failure, and b) eGFR at which dialysis was commenced.

## Methods

### Data source

The UK Renal Registry (UKRR) holds pseudonymised data for research purposes on all adults and children receiving long-term (>90 days) kidney replacement therapy (KRT) in the UK. eGFR results are collected on a monthly or quarterly basis, alongside information about KRT modality and patient characteristics. The authors did not have access to information that could identify individual participants during or after data collection for the purposes of this analysis.

### Study design

This was a retrospective cohort analysis using data collected and prospectively maintained by the UKRR. We identified all prevalent adult patients (≥18 years) with a failed kidney transplant between 01/2012 and 12/2021 receiving treatment in England, Wales and Northern Ireland (the UKRR does not include eGFR data for Scotland). We excluded patients who had opted out of research via the NHS National Data Opt Out.

There are no national or international consensus definitions of ‘kidney transplant failure’. We chose an eGFR value of <15 ml/min/1.73 m^2^ to represent transplant ‘failure’, since this value defines kidney failure in native kidney disease [11]. Data was first accessed and identified for this analysis in July 2024.

### Kidney treatment status

We classified a change in KRT modality after transplant failure as the first treatment status recorded of: i) re-transplantation, ii) haemodialysis (HD), iii) peritoneal dialysis (PD) or iv) death. Those classified as re-transplanted were, by definition, *pre-emptively* re-transplanted because we recorded treatment status based on the *first* new KRT modality detected post-failure. As in native kidney failure, there is variation in the time to KRT modality change after a patient reaches kidney transplant failure as defined as eGFR < 15 ml/min/1.73m^2^. For example, some people will be highly symptomatic, have rapidly declining function and change KRT within a year of transplant failure; others may not undergo a change in KRT treatment for many years, and have stable function and few symptoms despite an eGFR < 15 ml/min/1.73 m^2^.

Receipt of conservative kidney management, defined as planned palliative care without dialysis, is not captured by the UKRR [12]. We classified those alive without changing KRT modality as v) alive without new KRT, subclassified as vi) “managed conservatively” if eGFR < 7.5 ml/min/1.73 m^2^ with no future dialysis initiation captured in our follow up. We chose an eGFR of <7.5 ml/min/1.73 m^2^ using our 25^th^ percentile for last eGFR prior to dialysis initiation (7.7 ml/min/1.73 m^2^) and rounding down to the nearest 0.5 ml/min/1.73 m^2^ eGFR.

We followed individuals for up to five years after their index date of transplant failure. Treatment status was reported by each year post-failure.

We performed further analysis of treatment status by one-year. We described treatment status by one-year following transplant failure overall and by: age, sex, ethnicity, socioeconomic group, primary renal disease (PRD) and treatment centre type.

### Analysis

We recorded the last available eGFR prior to dialysis start, allowing results from a maximum of 3 months pre-dialysis. Some centres submit data monthly while others submit quarterly, with transplant centres more likely to send data monthly. Therefore, data frequency (monthly/quarterly) was included in our final pre-dialysis eGFR model, as this could confound the association between centre type and eGFR. We calculated eGFR using the CKD-EPI 2009 equation which includes ‘adjustment’ for ethnicity.

Sex is recorded as entered by the kidney unit. Ethnicity is recorded as per the Office for National Statistics categories [13]. PRD is recorded according to the European Renal Association-European Dialysis and Transplant Association (ERA-EDTA) PRD groupings [14]. The Index of Multiple Deprivation (IMD) provides a national-specific, area-based measure of relative socioeconomic deprivation according to postcode, which we categorised into quintiles [15,16].

We defined centres (hospitals) as transplanting or non-transplanting based on whether they perform kidney transplant surgery. We performed an initial analysis including clinic type as a variable, based on our 2022 UK practice patterns survey [9]. Clinic type was not included as a study variable in our final analyses as it correlated with centre type (S1 Table) and no additional associations were identified. Unadjusted funnel plots outlining centre variation in treatment status by one-year post-failure were created.

### Sensitivity analyses

In 2021, recommendations to remove adjustments for ethnicity when calculating eGFR were implemented [14,17]. We present our findings with the inclusion of the ethnicity correction factor since this was the eGFR used by clinicians to inform treatment decision making during our analysis timeframe. However, regression analyses with the correction factor retrospectively removed were also performed.

### Statistical analyses

Normally distributed continuous variables are reported with means and standard deviations. Non-normally distributed continuous variables are reported with medians and interquartile ranges (IQR).

Univariable associations between patient characteristics and treatment status after transplant failure were tested using chi-squared tests for categorical variables.

Regression models were used to investigate the relationship between patient characteristics and centre type, with both i) treatment status by one-year post-failure and ii) eGFR prior to dialysis start.

We used multinomial logistic regression to investigate the relationship between patient characteristics and centre type with treatment status by one-year post-failure. People managed conservatively were grouped with those alive without new KRT, due to small numbers of the former. Results are presented as adjusted relative risk ratios (RRR) of a one-year treatment status (re-transplantation, death, haemodialysis or peritoneal dialysis) relative to remaining alive without new KRT. RRR are reported with 95% confidence intervals (CI) for the association of a patient characteristic or centre type with each treatment status. p-values are from F-tests for the association of a patient characteristic or centre type with overall treatment status.

Linear regression was used to investigate the relationships between patient characteristics and centre type with eGFR prior to dialysis start. eGFR was modelled as a continuous variable using log transformation to better satisfy modelling assumptions. Results were exponentiated and reported as percentage differences in pre-dialysis eGFR between comparison groups.

When investigating their relationships with treatment status and pre-dialysis eGFR, age, sex, ethnicity, IMD, PRD and centre type were mutually adjusted-for. Identification of these variables as potential confounders to the explored associations was undertaken *a priori*.

Between-centre variation was explored using multilevel models. Specifically, a random intercept term was added to the model to allow for clustering by centre. This model was chosen over a single level model if a likelihood ratio test demonstrated improved fit.

Regression results were reported with 95% CI throughout, with p-values from F-tests. p-values ≤0.05 were considered as indicating good evidence against the null hypothesis. All analyses were conducted in SAS v9.4. This paper was written with reference to STROBE guidance [18].

### Missing data

Patients lost to follow up before one year were excluded from all analyses. Patients with incomplete covariate data were excluded from regression analyses. Those who started dialysis within one year but did not have an eGFR recorded within 3 months of starting dialysis were excluded from the pre-dialysis eGFR regression analysis.

### Ethical considerations and approval

This study involved the analysis of de-identified data under UK Renal Registry Research Ethics Committee permissions for research. The UKRR holds permissions under s251 of the National Health Service (NHS) Act 2006, to gather, process, and share confidential patient information for the purposes of audit and research. The collection and analysis of the data for research were carried out under the ethics permissions granted to the UKRR by a Research Ethics Committee: 25/NE/0190. Patients who have opted out of all secondary uses of their confidential information via the National Data Opt-out were not included in the study cohort.

## Results

**Fig 1:**
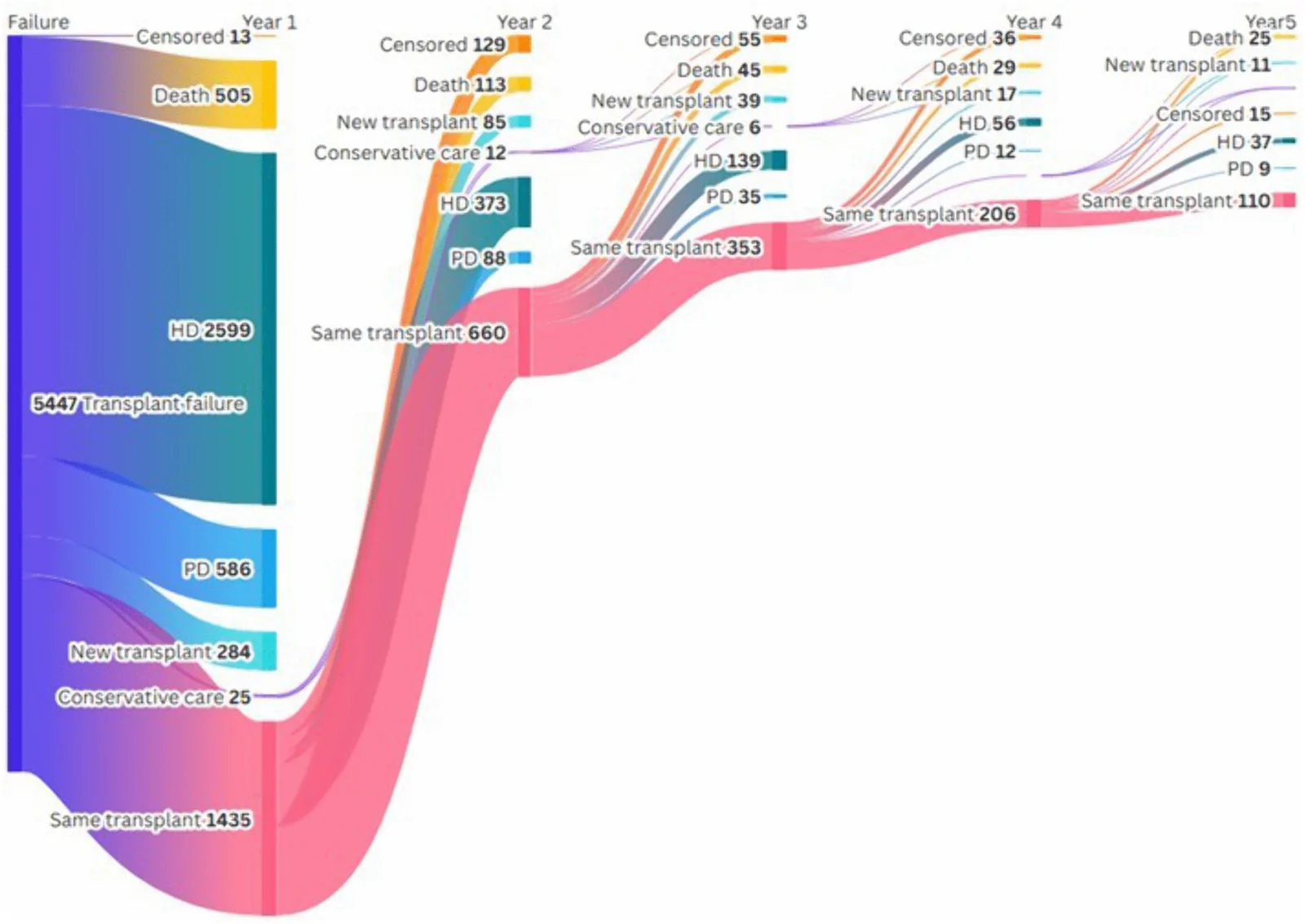
Sankey chart showing outcomes up to 5 years post-transplant failure. Sankey chart showing outcomes up to 5 years post-transplant failure (Censored: Lost to follow up, HD: Haemodialysis, PD: Peritoneal dialysis, Same transplant: Alive without new KRT).

**Fig 2:**
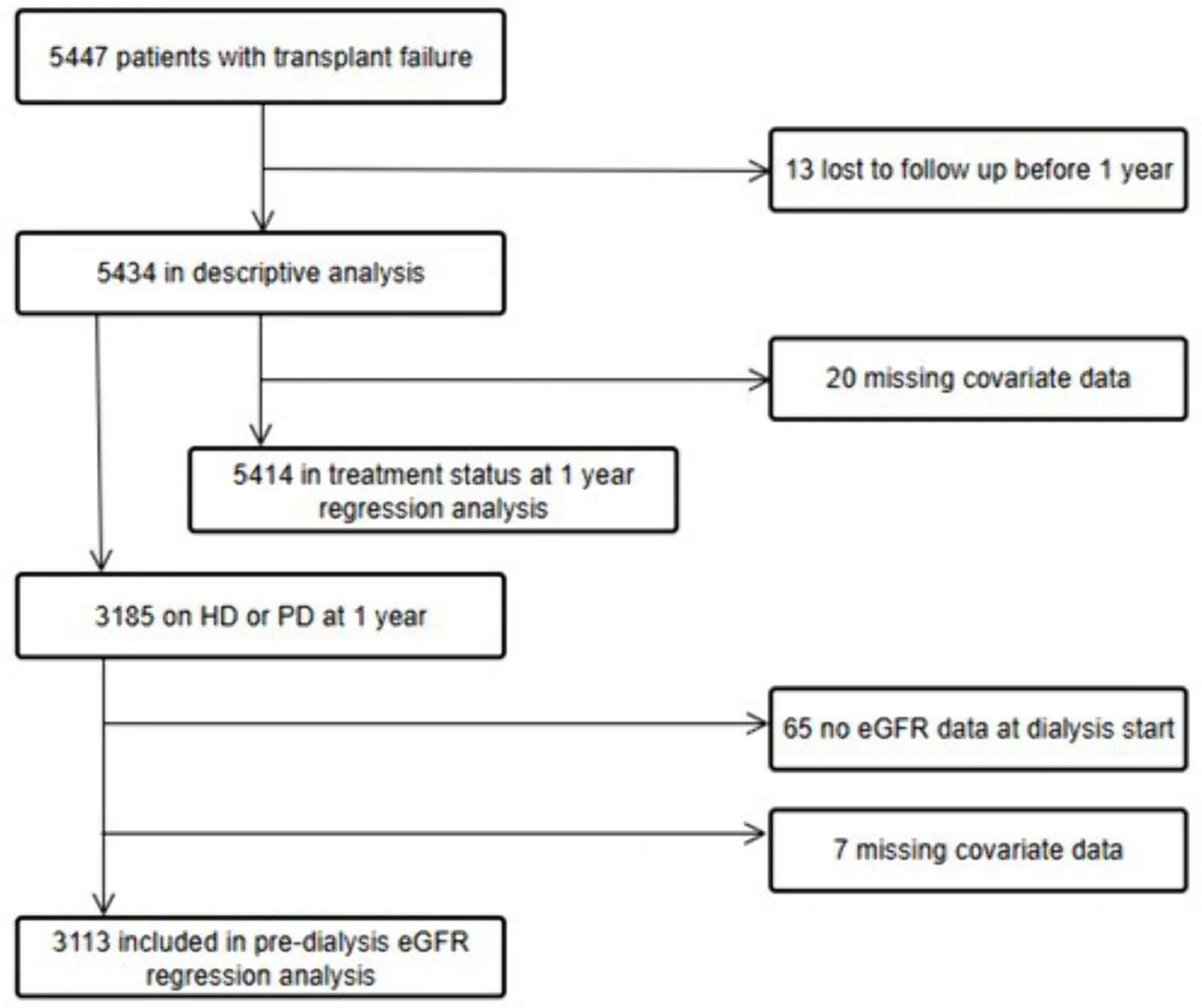
Data analysis flow chart.

5,447 people with transplant failure between 2012 and 2021 were identified. Their treatment statuses up to 5 years post-failure are presented in a Sankey diagram (Fig 1). Thirteen patients were lost to follow up before one year, leaving 5,434 in the descriptive analysis (Tables 1 and 2). Twenty-seven patients were removed from regression analyses due to missing covariate data. Sixty-five people had commenced dialysis within one year, but their eGFR at dialysis start wasn’t available, excluding them from the pre-dialysis eGFR analysis (Fig 2 and S2 Table).

**Table 1:** Treatment status by one-year post-transplant failure by patient characteristics.

		All	Death	HD	New transplant	PD	Same transplant	Conservative care	p-value
**All**	**N (%)**	5434 (100)	505 (9.3)	2599 (47.8)	284 (5.2)	586 (10.8)	1435 (26.4)	25 (0.5)	
**Age at failure**	**Median (IQR)**	55.4 (45.2,65.1)	68.1 (58.3,74.6)	53.2 (42.9,62.9)	45.2 (37.3,54.7)	51.2 (40.5,61.5)	58.2 (49.3,66.4)	54.6 (48.6,68.4)	<0.001
**Pre-dialysis eGFR**	**Median (IQR)**	9.6 (7.7,11.7)		9.7 (7.8,11.7)		9.1 (7.4,11.2)			0.001
		**N**	**N (row %)**	**N (row %)**	**N (row %)**	**N (row %)**	**N (row %)**	**N (row %)**	
**Sex**									
	**Male**	3015	251 (8.3)	1605 (53.2)	156 (5.2)	295 (9.8)	698 (23.1)	11 (0.4)	<0.001
	**Female**	2416	254 (10.5)	992 (41.1)	128 (5.3)	291 (12.0)	737 (30.5)	14 (0.6)	
**Ethnicity**									
	**Asian**	627	54 (8.6)	353 (56.4)	19 (3.0)	55 (8.8)	145 (23.2)	<5	<0.001
	**Black**	443	21 (4.8)	280 (63.3)	8 (1.8)	33 (7.5)	100 (22.6)	<5	
	**Mixed/Other**	147	7 (4.9)	86 (59.7)	6 (4.2)	12 (8.3)	33 (22.9)	3 (2.0)	
	**White**	4214	423 (10)	1880 (44.6)	251 (6.0)	486 (11.5)	1157 (27.4)	19 (0.5)	
**IMD quintile**									
	**1 (most deprived)**	1288	116 (9.0)	688 (53.4)	43 (3.3)	110 (8.5)	323 (25.1)	9 (0.7)	<0.001
	**2**	1192	104 (8.7)	604 (50.6)	49 (4.1)	122 (10.2)	309 (25.9)	5 (0.4)	
	**3**	1053	96 (9.2)	496 (47.3)	60 (5.7)	120 (11.4)	277 (26.4)	<5	
	**4**	991	105 (10.6)	436 (44.0)	70 (7.1)	109 (11.0)	265 (26.7)	6 (0.6)	
	**5 (least deprived)**	905	84 (9.3)	375 (41.4)	61 (6.7)	125 (13.8)	260 (28.7)	<5	
	**Missing**	2							
**PRD**									
	**Diabetic**	589	73 (12.5)	335 (57.2)	19 (3.2)	38 (6.5)	121 (20.6)	<5	<0.001
	**Glomerulonephritis**	1328	96 (7.2)	697 (52.4)	75 (5.6)	165 (12.4)	293 (22.0)	5 (0.4)	
	**Hypertensive**	376	39 (10.4)	204 (54.5)	6 (1.6)	29 (7.8)	96 (25.7)	<5	
	**Other**	1013	68 (6.7)	472 (46.7)	73 (7.2)	131 (13)	267 (26.4)	<5	
	**Polycystic**	539	76 (14.2)	204 (38.1)	12 (2.2)	41 (7.7)	202 (37.8)	<5	
	**Pyelonephritis**	788	59 (7.5)	333 (42.5)	54 (6.9)	102 (13)	235 (30.0)	<5	
	**Renovascular**	78	15 (19.2)	31 (39.7)	3 (3.8)	5 (6.4)	24 (30.8)	<5	
	**Uncertain**	702	75 (10.7)	316 (45.3)	42 (6.0)	75 (10.7)	190 (27.2)	<5	
	**Missing**	18							

*p-*value from chi-squared tests for categorical variables, Kruskal-Wallis tests for continuous variables. ‘Conservative care’ and ‘Same transplant’ were grouped together for testing due to small numbers on conservative care. Where cell numbers are shown as <5, percentages are calculated excluding that cell. HD: Haemodialysis, PD: Peritoneal dialysis, Same transplant: alive without new KRT, IMD: Index of Multiple Deprivation, PRD: Primary renal disease.

**Table 2:** Treatment status by one-year post transplant failure by centre characteristics.

		All	Death	HD	New transplant	PD	Same transplant	Conservative care	p-value
**All**	**N (%)**	5434 (100)	505 (9.3)	2599 (47.8)	284 (5.2)	586 (10.8)	1435 (26.4)	25 (0.5)	
		**N**	**N (row %)**	**N (row %)**	**N (row %)**	**N (row %)**	**N (row %)**	**N (row %)**	
**Centre type**	**Tx centre**	3603	327 (9.1)	1721 (47.7)	208 (5.8)	376 (10.4)	956 (26.5)	18 (0.5)	0.085
	**Non-Tx centre**	1828	178 (9.7)	878 (48.0)	76 (4.2)	210 (11.5)	479 (26.2)	7 (0.4)	

*p-*value from chi-squared tests for categorical variables. ‘Conservative care’ and ‘Same transplant’ were grouped together for regression analyses due to small numbers on conservative care. HD: Haemodialysis, PD: Peritoneal dialysis, Same transplant: alive without new KRT, Tx: Transplanting.

By one year following transplant failure, the first outcome for 9.3% (505) of people was death. Most people first started HD (2599, 47.8%), one quarter were alive without new KRT (1435, 26.4%). Smaller proportions first received PD (586, 10.8%) or had been pre-emptively re-transplanted (284, 5.2%). The lowest proportion were assumed to be receiving conservative management (25, 0.5%).

Median pre-dialysis eGFR was 9.6 ml/min/1.73m^2^ (IQR 7.7–11.7); 9.7 ml/min/1.73m^2^ for those commencing HD versus 9.1 ml/min/1.73m^2^ pre-PD.

### Centre type

We did not find strong evidence that centre type (transplanting versus non-transplanting) was associated with one-year treatment status (*p* = 0.137. Table 3). However, there was weak evidence of reduced likelihood of re-transplantation amongst people managed in non-transplanting compared to transplanting centres (RRR 0.66, 0.43–1.00).

**Table 3:** Relative risk ratios from multivariable multilevel regression for one-year treatment status relative to remaining on the same transplant.

		Death vs Same transplant	HD vs Same transplant	New transplant vs Same transplant	PD vs Same transplant	p-value
**Variable**		**Relative Risk Ratio (95% confidence intervals)**				
**Centre type**	**Non-Tx centre vs Tx centre**	1.02 (0.80, 1.32)	1.13 (0.95, 1.35)	0.66 (0.43, 1.00)	1.08 (0.81, 1.44)	0.137
**Age at failure**	**1 year Increase**	1.07 (1.06, 1.08)	0.97 (0.97, 0.98)	0.94 (0.93, 0.95)	0.97 (0.96, 0.97)	<0.001
**Sex**	**Male vs Female**	0.95 (0.76, 1.17)	1.68 (1.47, 1.92)	1.39 (1.06, 1.81)	1.09 (0.89, 1.33)	<0.001
**Ethnicity**						
	**Asian vs White**	0.98 (0.69, 1.40)	1.40 (1.12, 1.74)	0.57 (0.34, 0.96)	0.87 (0.62, 1.24)	<0.001
	**Black vs White**	0.50 (0.29, 0.84)	1.53 (1.18, 1.98)	0.41 (0.20, 0.87)	0.83 (0.54, 1.28)	
	**Mixed/Other vs White**	0.55 (0.24, 1.28)	1.34 (0.89, 2.02)	0.68 (0.28, 1.69)	0.78 (0.40, 1.54)	
**IMD quintile**	**1 quintile increase (worsening deprivation)**	1.07 (0.99, 1.15)	1.04 (0.99, 1.09)	0.78 (0.71, 0.86)	0.88 (0.82, 0.94)	0.001
**PRD**						
	**Diabetic vs Glomerulonephritis**	1.97 (1.34, 2.88)	1.20 (0.93, 1.54)	0.69 (0.39, 1.20)	0.58 (0.38, 0.88)	<0.001
	**Hypertensive vs Glomerulonephritis**	1.17 (0.74, 1.84)	0.85 (0.64, 1.13)	0.36 (0.15, 0.86)	0.62 (0.39, 0.99)	
	**Polycystic vs Glomerulonephritis**	0.82 (0.57, 1.18)	0.57 (0.44, 0.73)	0.35 (0.18, 0.67)	0.44 (0.30, 0.65)	
	**Pyelonephritis vs Glomerulonephritis**	0.87 (0.59, 1.27)	0.60 (0.48, 0.75)	0.71 (0.47, 1.07)	0.69 (0.50, 0.94)	
	**Renovascular vs Glomerulonephritis**	1.24 (0.61, 2.53)	0.65 (0.37, 1.14)	0.72 (0.20, 2.60)	0.48 (0.18, 1.28)	
	**Other vs Glomerulonephritis**	0.88 (0.61, 1.26)	0.71 (0.57, 0.87)	0.77 (0.52, 1.13)	0.74 (0.55, 1.00)	
	**Uncertain vs Glomerulonephritis**	1.13 (0.79, 1.64)	0.7 (0.56, 0.88)	1.02 (0.66, 1.57)	0.72 (0.52, 1.01)	

All variables (Centre type, age, sex, ethnicity, IMD quintile and primary renal disease) mutually adjusted for. *p-*values from F tests. HD: Haemodialysis, PD: Peritoneal dialysis, Same transplant: alive without new KRT, Tx: Transplanting, IMD: Index of Multiple Deprivation, PRD: Primary renal disease.

After adjusting for patient characteristics and centre type, centre variation remained. The multilevel model accounting for centre variation was a better fit than a single level model (*p* < 0.001 comparing fit to model with no centre variation). Unadjusted individual centre variation is presented in Funnel plot form (S1 Fig).

### Associations between patient characteristics and one-year treatment status

Age, sex, ethnicity, socioeconomic deprivation and PRD were all associated with treatment status by one-year (*p ≤* 0.0001 in both univariable (Table 1) and multivariable analyses (Table 3)).

### Age

Increasing age was associated with higher likelihood of death after transplant failure (RRR per one-year increase 1.07, 1.06–1.08) and lower likelihood of re-transplantation relative to remaining alive without new KRT (RRR 0.94, 0.93–0.95).

### Sex

Males were more likely to have transitioned onto another treatment after transplant failure as opposed to remaining alive without new KRT. Specifically, males were more likely than females to be re-transplanted after transplant failure (RRR 1.39, 1.06–1.81) and to be receiving HD (RRR 1.68, 1.47–1.92).

### Ethnicity

Black people were 59% less likely (RRR 0.41, 0.20–0.87) and Asian people 43% less likely (RRR 0.57, 0.34–0.96) than White people to have received a new transplant, relative to their likelihood of remaining alive without new KRT. Both Black and Asian people were more likely to have transitioned to HD, compared with White people (Black versus White RRR 1.53, 1.18–1.98. Asian versus White RRR 1.40, 1.12–1.74). No strong evidence of an association between ethnicity and likelihood of transitioning to PD was observed. Black people were 50% less likely to die than remain alive without new KRT, when compared with White people (Black patient deaths = 21/443, White patient deaths = 423/4214; RRR 0.50, 0.29–0.84), while there were no differences between Asian and White people (RRR 0.98, 0.69–1.40).

Sensitivity analyses performed with removal of the ethnicity correction factor (Tables 4 and 5) showed a larger reduction in the likelihood of re-transplantation for Black compared to White people (RRR 0.21, 0.10–0.43). When removing the ethnicity correction factor, Black people appeared less likely to commence either type of dialysis compared to White people (HD RRR 0.77, 0.62–0.97. PD RRR 0.40, 0.26–0.60) and their likelihood of death relative to White people reduced further (RRR 0.32, 0.20, 0.51).

**Table 4:** Relative risk ratios from multivariable multilevel regression for one-year treatment status relative to remaining on the same transplant.

		Death vs Same transplant	HD vs Same transplant	New transplant vs Same transplant	PD vs Same transplant	p-value
**Variable**		**Relative risk ratio (95% confidence intervals)**				
**Centre type**	**Non-Tx centre vs Tx centre**	1.01 (0.79, 1.3)	1.12 (0.95, 1.33)	0.65 (0.43, 0.99)	1.09 (0.82, 1.46)	0.119
**Age at failure**	**1 year Increase**	1.07 (1.06, 1.08)	0.97 (0.97, 0.98)	0.94 (0.93, 0.95)	0.97 (0.96, 0.97)	<0.001
**Sex**	**Male vs Female**	0.95 (0.77, 1.17)	1.70 (1.49, 1.94)	1.39 (1.06, 1.81)	1.09 (0.90, 1.33)	<0.001
**Ethnicity**						
	**Asian vs White**	0.98 (0.68, 1.39)	1.39 (1.12, 1.74)	0.56 (0.33, 0.95)	0.86 (0.61, 1.23)	<0.001
	**Black vs White**	0.32 (0.20, 0.51)	0.77 (0.62, 0.97)	0.21 (0.10, 0.43)	0.40 (0.26, 0.60)	
	**Mixed/Other vs White**	0.54 (0.23, 1.27)	1.34 (0.89, 2.01)	0.68 (0.28, 1.68)	0.78 (0.40, 1.54)	
**IMD quintile**	**1 quintile increase (worsening deprivation)**	1.07 (0.99, 1.16)	1.04 (0.99, 1.09)	0.79 (0.72, 0.87)	0.89 (0.83, 0.96)	<0.001
**PRD**						
	**Diabetic vs Glomerulonephritis**	1.85 (1.27, 2.70)	1.17 (0.92, 1.49)	0.65 (0.37, 1.14)	0.54 (0.36, 0.82)	<0.001
	**Hypertensive vs Glomerulonephritis**	1.25 (0.80, 1.95)	0.89 (0.68, 1.18)	0.37 (0.16, 0.89)	0.66 (0.41, 1.04)	
	**Polycystic vs Glomerulonephritis**	0.82 (0.57, 1.18)	0.59 (0.46, 0.75)	0.35 (0.18, 0.67)	0.45 (0.30, 0.66)	
	**Pyelonephritis vs Glomerulonephritis**	0.88 (0.60, 1.28)	0.60 (0.49, 0.75)	0.69 (0.46, 1.03)	0.68 (0.50, 0.93)	
	**Renovascular vs Glomerulonephritis**	1.26 (0.62, 2.55)	0.67 (0.39, 1.18)	0.73 (0.20, 2.63)	0.49 (0.18, 1.31)	
	**Other vs Glomerulonephritis**	0.87 (0.61, 1.25)	0.72 (0.59, 0.88)	0.76 (0.52, 1.11)	0.72 (0.54, 0.97)	
	**Uncertain vs Glomerulonephritis**	1.17 (0.81, 1.68)	0.73 (0.58, 0.91)	1.03 (0.67, 1.59)	0.74 (0.53, 1.04)	

All variables (Centre type, age, sex, ethnicity, IMD quintile and primary renal disease) mutually adjusted for. *Black ethnicity correction factor removed.* *p-*values from F tests. HD: Haemodialysis, PD: Peritoneal dialysis, Same transplant: alive without new KRT, Tx: Transplanting, IMD: Index of Multiple Deprivation, PRD: Primary renal disease.

**Table 5:** Percentage difference in eGFR associated with patient characteristics, based on multivariable multilevel regression model adjusting for all listed variables, including adjustment for eGFR data source.

Variable		Percentage difference in pre-dialysis eGFR (95% confidence intervals)	p-value
**Centre type**	**Non-Tx centre vs Tx centre**	+6.7% (+2.3%, + 11.4%)	0.003
**Age at failure**	**1 year increase**	+0.2% (+0.1%, + 0.3%)	<0.001
**Sex**	**Male vs Female**	+5.1% (+2.8%, + 7.4%)	<0.001
**Ethnicity**			
	**Asian vs White**	−2.7% (−6.0%, + 0.7%)	<0.001
	**Black vs White**	−11.9% (−15.3%, −8.2%)	
	**Mixed/Other vs White**	−3.9% (−9.8%, + 2.4%)	
**IMD quintile**	**1 quintile increase**	+0.5% (−0.3%, + 1.3%)	0.186
**PRD**			
	**Diabetic vs Glomerulonephritis**	+7.6% (+3.6%, + 11.6%)	<0.001
	**Hypertensive vs Glomerulonephritis**	−3.1% (−7.4%, + 1.3%)	
	**Polycystic vs Glomerulonephritis**	+1.8% (−2.5%, + 6.3%)	
	**Pyelonephritis vs Glomerulonephritis**	+4.6% (+1.0%, + 8.4%)	
	**Renovascular vs Glomerulonephritis**	−8.0% (−16.9%, + 1.6%)	
	**Other vs Glomerulonephritis**	+4.3% (+1.0%, + 7.7%)	
	**Uncertain vs Glomerulonephritis**	+0.3% (−3.3%, + 4.0%)	
**eGFR Data Frequency**	**Monthly vs Quarterly eGFR Data**	−9.6% (−12.9%, −7.1%)	<0.001

*p-*values from F tests. Tx: Transplanting, IMD: Index of Multiple Deprivation, PRD: Primary renal disease. *Black ethnicity correction factor removed.*

### Socioeconomic deprivation

Higher area-level deprivation was associated with a lower likelihood of both re-transplantation (RRR 0.78, 0.71–0.86 per one quintile increase in deprivation) and PD (RRR 0.88, 0.82–0.94) by one year post transplant failure, versus remaining alive without new KRT.

### Primary renal disease

People with diabetic nephropathy were 97% more likely than people with glomerulonephritis to have died than to have remained alive by one year post transplant failure without new KRT (RRR 1.97, 1.34–2.88).

### Pre-Dialysis eGFR

Pre-dialysis eGFR was 7.1% higher for those managed at non-transplanting centres compared with transplanting centres (Percentage difference in eGFR: + 7.1%, + 2.6% to +11.9%. *p* = 0.002. Table 6). People with diabetes as their PRD, had an 8.5% higher pre-dialysis eGFR than those with glomerulonephritis (Percentage difference in eGFR: + 8.5%, + 4.6% to +12.5%.).

**Table 6:** Percentage difference in eGFR associated with patient and centre characteristics, based on multivariable multilevel regression model adjusting for all listed variables, including adjustment for eGFR data source.

Variable		Percentage difference in pre-dialysis eGFR (95% confidence intervals)	p-value
**Centre type**	**Non-Tx centre vs Tx centre**	+7.1% (+2.6%, + 11.9%)	0.002
**Age at failure**	**1 year increase**	+0.2% (+0.1%, + 0.3%)	<0.001
**Sex**	**Male vs Female**	+5.5% (+3.3%, + 7.8%)	<0.001
**Ethnicity**			
	**Asian vs White**	−2.9% (−6.1%, −0.1%)	0.026
	**Black vs White**	−5.3% (−9.0%, −1.5%)	
	**Mixed/Other vs White**	−3.9% (−9.7%, + 2.3%)	
**IMD quintile**	**1 quintile increase (worsening deprivation)**	+0.6% (−0.2%, + 1.4%)	0.135
**PRD**			
	**Diabetic vs Glomerulonephritis**	+8.5% (+4.6%, + 12.5%)	<0.001
	**Hypertensive vs Glomerulonephritis**	−2.2% (−6.5%, + 2.2%)	
	**Polycystic vs Glomerulonephritis**	+2.2% (−2.1%, + 6.7%)	
	**Pyelonephritis vs Glomerulonephritis**	+5.4% (+1.8%, + 9.2%)	
	**Renovascular vs Glomerulonephritis**	−7.1% (−15.9%, + 2.6%)	
	**Other vs Glomerulonephritis**	+4.3% (0.0%, + 7.6%)	
	**Uncertain vs Glomerulonephritis**	+1.2% (−2.4%, + 4.9%)	
**eGFR Data Frequency**	**Monthly vs Quarterly eGFR Data**	−9.5% (−11.9%, −7.1%)	<0.001

*p-*values from F tests. Tx: Transplanting, IMD: Index of Multiple Deprivation, PRD: Primary renal disease.

## Discussion

In this large national registry analysis, we found strong evidence of inequalities in access to pre-emptive re-transplantation; more likely amongst White people, those living in the least deprived areas and males. Differences in management between those in transplanting and non-transplanting centres was observed, with dialysis commenced earlier in non-transplanting centres and a signal towards lower likelihood of re-transplantation in non-transplanting centres identified. Worsening deprivation was not only associated with lower likelihood of re-transplantation, but also with a lower likelihood of PD. Death in those with diabetic nephropathy was twice as likely compared to in those with glomerulonephritis.

Although UK-based, prospective, observational research assessing inequalities in access to first transplantation has been done [19], to our knowledge, our study is the first to explore inequalities in access to pre-emptive re-transplantation in the UK. Black people have a 34% lower likelihood of receiving a first transplant compared with White people [20]; we found a 59% lower likelihood of pre-emptive re-transplantation, widening ethnic disparity at re-transplantation. In the US, the likelihood of first transplantation is 32% lower in Black people compared with White people and 39% lower at pre-emptive re-transplantation, similarly suggesting worsening inequalities at re-transplantation [21,22].

One explanation for a widening disparity is cumulative immunological disadvantage; sensitisation from a first transplant is likely to have a greater impact on likelihood of future transplant offers in those people with a blood group and tissue type less commonly found in the donor population. In 2024/25, Minority Ethnic groups comprised 7% of deceased kidney donors in the UK, but 35% of the active transplant list [1]. Deceased donor kidney allocation is based on ABO-compatibility and human leukocyte antigen matching, which is more likely if donors and recipients are from the same ethnic group. Changes made to the national kidney allocation scheme in 2019 resulted in prioritisation of potential recipients who are immunologically hard to match to a UK organ donor [23]. A future FAKTOR output will analyse re-transplantation access in greater depth, comparing data pre- and post-allocation scheme changes and incorporating immunological matchability metrics.

Additional proposed barriers to equal transplantation access between ethnic groups include religious beliefs, misperceptions and distrust of the medical community [24,25]. In a cohort already transplanted, these traditionally cited barriers to transplantation receipt should be less applicable to the potential recipient. However, they may still impact living donation likelihood from within a potential recipient’s family and community. Qualitative research exploring barriers during re-transplantation work-up from the perspectives of people from Minority Ethnic groups with failed transplants is required.

Our finding of a reduced likelihood of pre-emptive re-transplantation with higher socioeconomic deprivation is consistent with previous international research in first kidney transplants after native kidney failure [26–28] and re-transplantation after transplant failure. In the US, pre-emptive re-transplantation is 51% less likely in unemployed people compared to those working full-time [21]. Similarly, in Australia and New Zealand, people in the most socioeconomically disadvantaged areas are 30% less likely to receive repeat transplants than those living in the least disadvantaged areas [29]. One barrier to transplantation is waiting list access, known to be lower with higher socioeconomic deprivation in the UK [19,30], though no listing data relating to the failed transplant cohort exists.

Males were more likely to have transitioned to dialysis, or have received a new transplant, than females. In native kidney failure, the subject of why a consistently higher proportion of males have been found to initiate KRT, despite higher CKD prevalence in females remains uncertain [31]. One reason may be due to the higher rate of renal function decline in males, necessitating a more urgent change in KRT once identified as having transplant failure [32]. Another may be higher uptake of conservative kidney management in females, with observational Australian data exploring patterns in conservative kidney management giving support to this theory [33]. Our definition of conservative kidney management was an estimate which inaccurately captured this cohort and therefore was insufficient to identify any definitive differences in conservative kidney management uptake between sexes.

Re-listing patterns have not been explored in the UK’s failed transplant cohort but may be relevant to understanding why re-transplantation favours males. In prospective UK data regarding initial transplantation, females on dialysis were 18% less likely to be listed [19]. However, that data found no difference in pre-emptive listing rates and the most recent UKRR analyses found males and females were as likely to be wait-listed and transplanted for the first time once they have started KRT [34].

We found a lower likelihood of death in Black people with transplant failure compared with White people. Whilst this warrants further investigation, the small number of deaths amongst the small Black cohort (Black patient deaths = 21/443, White patient deaths = 423/4214) makes interpretation of this finding challenging, especially when considering the higher prevalence of hypertension, diabetes and coronary artery disease in this population which would be expected to increase mortality [35,36]. There is some support for our finding from previous UK and US registry analyses reporting a survival advantage for Black people on dialysis [37,38]: however, these findings are likely to affected by selection bias resulting from a greater proportion of transplant eligible Black people remaining on dialysis compared to transplant eligible White people. An analysis based on registry data is unable to disentangle potential causative factors driving mortality and prospective research gathering additional data about co-morbidities and lifestyle factors is needed in the failed transplant cohort.

We found no strong evidence that centre type (transplanting vs non-transplanting) was associated with kidney treatment status by one-year after transplant failure. However, patients managed in transplant centres had lower pre-dialysis eGFRs (i.e., started dialysis later), and there was weak evidence of a reduced likelihood of re-transplantation in non-transplanting centres. In the UK, patients registered at transplanting centres are reported to be three times more likely to be pre-emptively listed for first transplantation [19]. It is not known if a similar association exists at listing for re-transplantation; centre variation will be assessed in our re-listing analysis. In the US, inter-centre variation has been observed, with rates of pre-emptive listing for re-transplantation differing between transplant centres [6,39].

### Strengths and limitations

This large registry analysis is the first analysis of outcomes following transplant failure in the UK. A methodological strength is our ability to capture data from centres in England, Wales and Northern Ireland, including people from marginalised groups who are underrepresented and poorly recruited into research studies [40].

Conclusions about the reasons for differences in access to re-transplantation are limited without data on tissue type, immunological sensitisation, re-listing and more detailed comorbidity metrics aside from primary renal disease. We plan to undertake a future analysis of linked UKRR and UK Transplant registry data to explore re-listing and re-transplantation patterns in the failed transplant population, alongside matchability scores, sensitisation metrics and comorbidities. Time to waitlisting after transplant failure, time to re-transplantation once listed and likelihood of living versus deceased kidney transplantation will be assessed. We hope this future work will increase our understanding of our observed ethnic, socioeconomic and sex-related inequalities.

As described, we classified individuals with eGFR < 15 ml/min/1.73 m^2^ as having transplant failure, although some may have had stable but poor kidney function without evidence of decline. As a result, our cohort includes some individuals with no clinical need to transition to another KRT modality, likely leading to an overestimation of the proportion alive with a “failed” transplant.

Cumulative duration of kidney replacement therapy may influence outcomes following transplant failure. Because some people began kidney replacement therapy for their native kidney failure outside of the UK, or prior to a centre joining the renal registry, we lack sufficient data completeness to adjust for this.

Finally, conservative care is not yet recorded in registry data. Our eGFR cut-off to capture this cohort may be an inaccurate measure of this group. Those who died may have been managed conservatively before death, and those with an eGFR < 7.5ml/min/1.73m^2^ may still change KRT modality at a later timepoint. This highlights the need for improved definitions of conservative care and CKD stage in real world registry data, which would ideally be consistent between countries to enable like for like comparisons.

We highlight strong evidence of inequalities in access to pre-emptive re-transplantation in the UK according to patient characteristics, favouring people of White ethnicity, those living in the least deprived areas and males. Future FAKTOR outputs will explore re-transplantation and re-listing practices in greater depth, as well as causes of death in the failed transplant cohort.

## Supporting information

S1 Fig Unadjusted funnel plots showing centre variation in first treatment status by one-year post-failure. (PDF)

S1 Table Clinic type in transplanting vs non-transplanting centres. (PDF)

S2 Table Excluded patients, including patient and centre characteristics. (PDF)
