# Supplementary material for: Inequalities in outcomes after kidney transplant failure in the UK
Source: PLoS One. 2026 Aug 26;21(8):e0356769. doi: 10.1371/journal.pone.0356769 (PMC13514003; doi:10.1371/journal.pone.0356769)
Supplement: Supplementary file 1 [file pone.0356769.s001.pdf]

Supplementary Figure 1: Unadjusted funnel plots showing centre variation in first treatment status by one-year post-failure.

1a: Percentage whose first treatment status was death

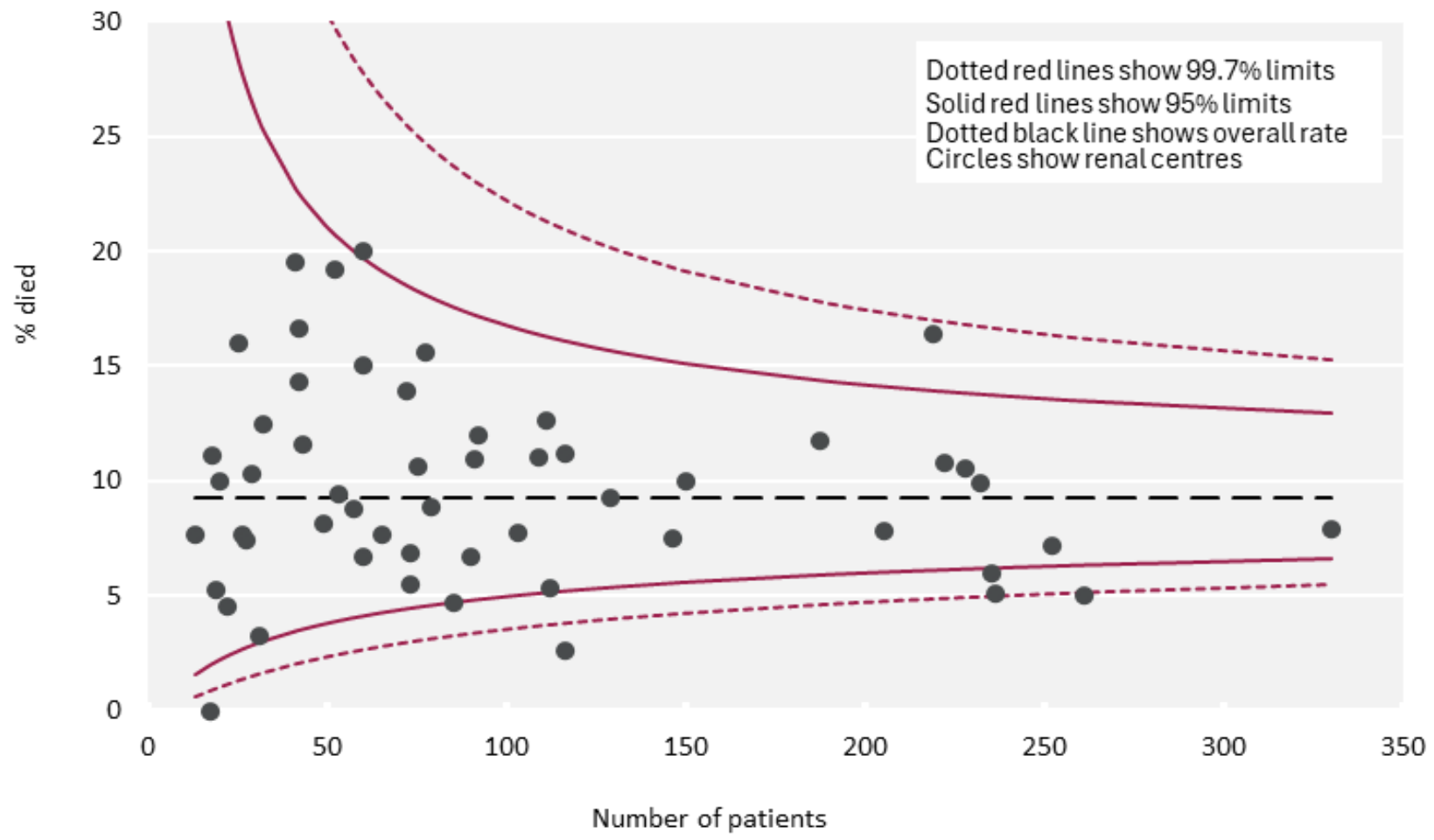

1b: Percentage whose first treatment status was dialysis

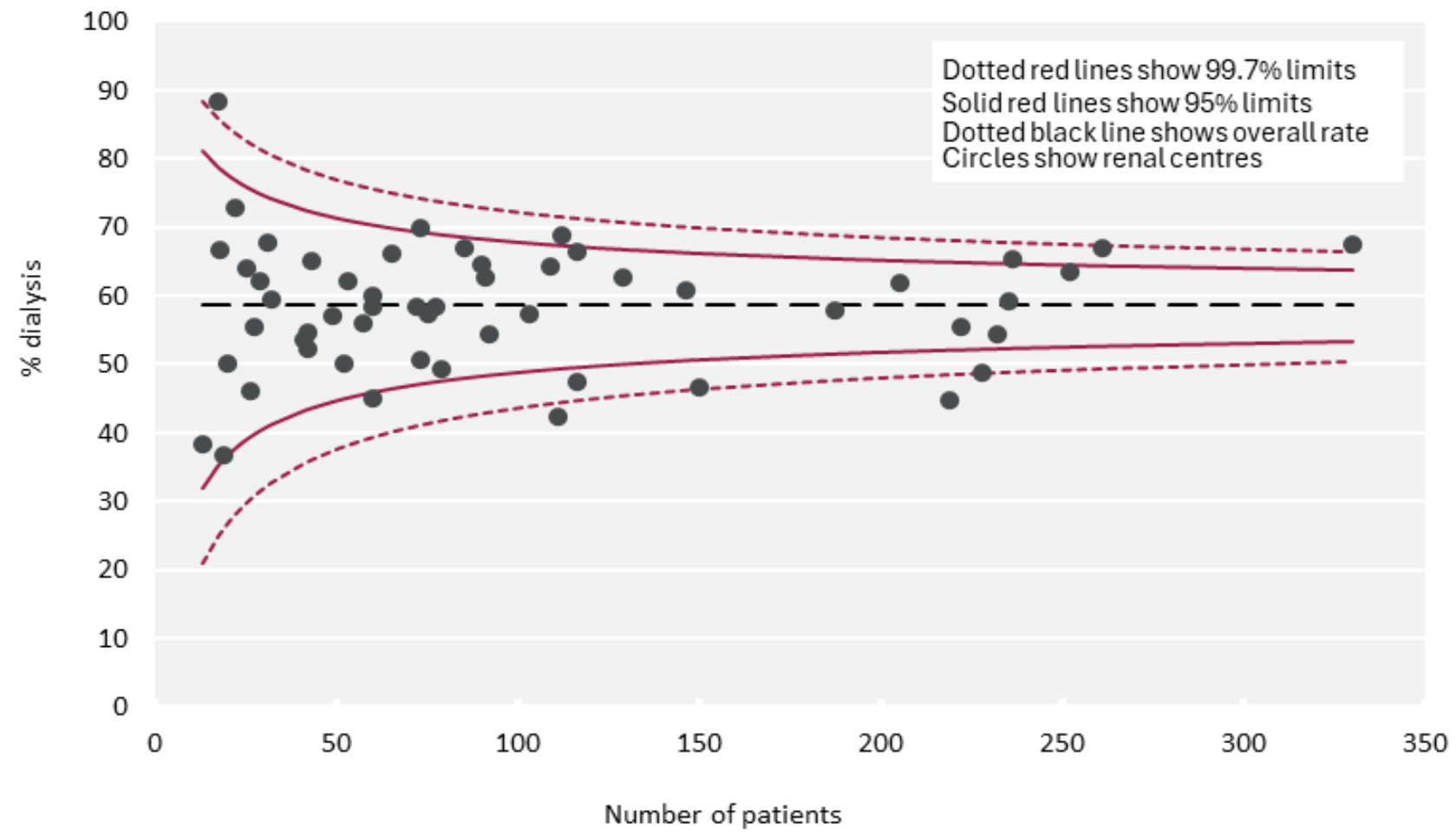

1c: Percentage whose first treatment status was re-transplantation

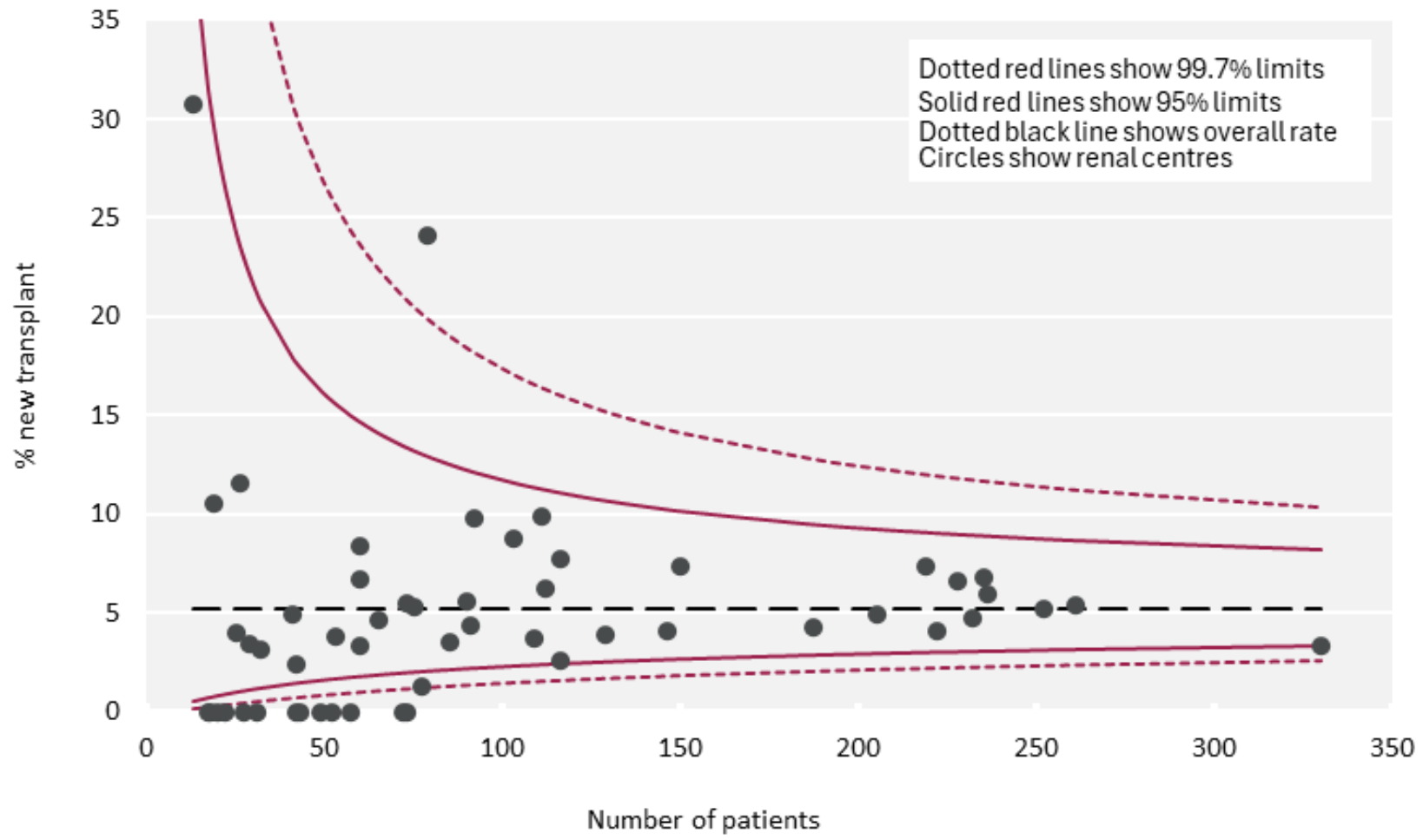

1d: Percentage alive without new kidney replacement therapy

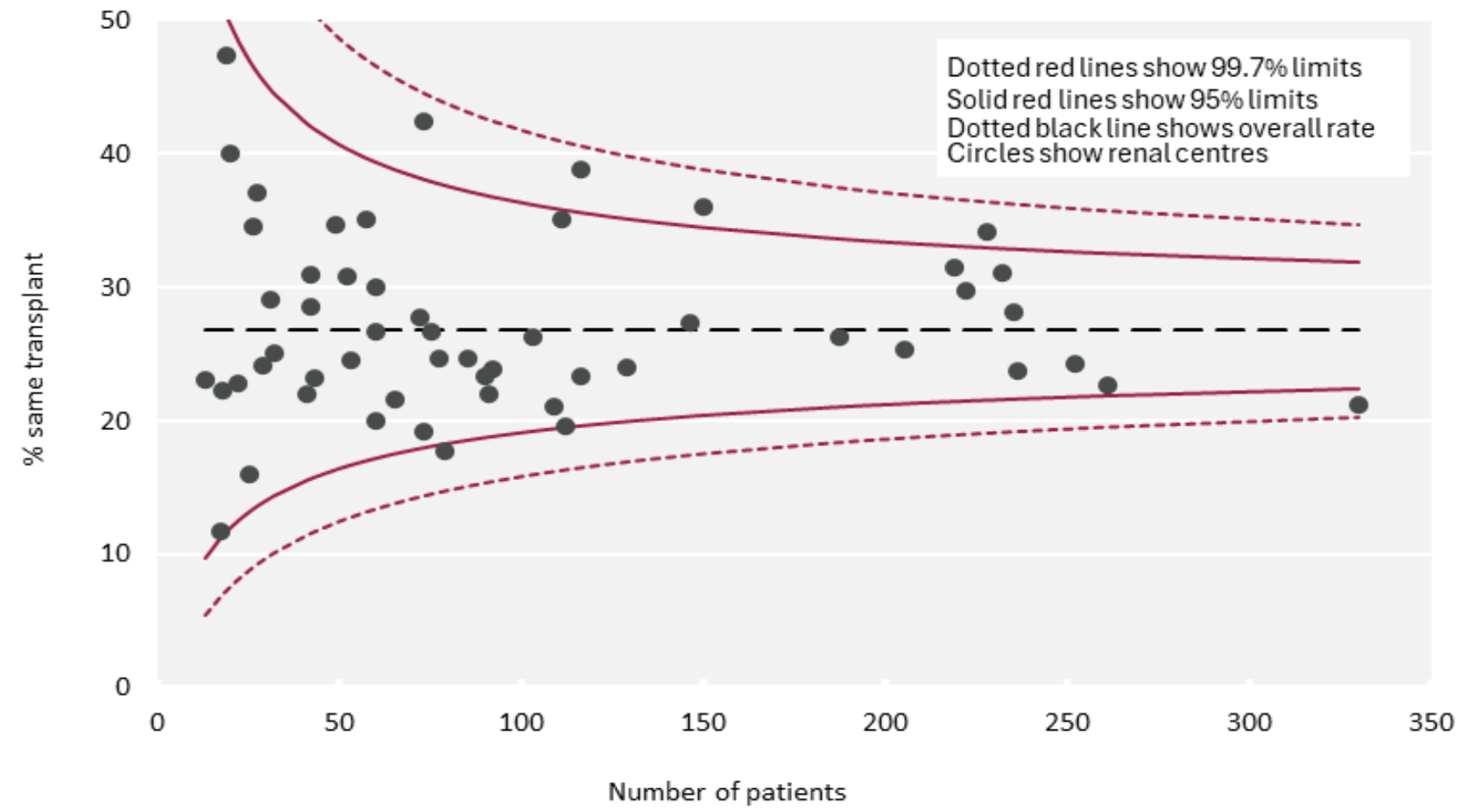

Centres with <10 patients were excluded for all funnel plots.
