# Supplementary material for: Inequalities in outcomes after kidney transplant failure in the UK
Source: PLoS One. 2026 Aug 26;21(8):e0356769. doi: 10.1371/journal.pone.0356769 (PMC13514003; doi:10.1371/journal.pone.0356769)
Supplement: Supplementary file 2 [file pone.0356769.s002.pdf]

Supplementary Table 1: Clinic type in Transplanting vs Non-Transplanting Centres

**Clinic Type**	**Non-Transplanting Centre**	**Transplanting Centre**	**Total**
General Low Clearance Clinic	1 (4.4%)*	6 (28.6%)	7
General Transplant Clinic	18 (78.3%)	4 (19.1%)	22
Low Clearance Transplant Clinic	2 (8.7%)	9 (42.9%)	11
Other	2 (8.7%)	2 (9.5%)	4
**Total**	23	21	44

**Number (Percentage clinic type within centre type).*
