# Supplementary material for: Inequalities in outcomes after kidney transplant failure in the UK
Source: PLoS One. 2026 Aug 26;21(8):e0356769. doi: 10.1371/journal.pone.0356769 (PMC13514003; doi:10.1371/journal.pone.0356769)
Supplement: Supplementary file 3 [file pone.0356769.s003.pdf]

Supplementary Table 2: Excluded patients, including patient and centre characteristics

		Excluded from 1 year treatment status regression analysis[1]		Excluded from pre-dialysis eGFR regression analysis[2]	
		N	%	N	%
**All**		33		72	
**Age at failure**	**Median**	57.1		49.8	
	**Lower quartile**	45.5		41.6	
	**Upper quartile**	63.8		60.7	
**Status at 1 year**					
**Censored**		13	39		
**Conservative care**		<5			
**Death**		<5			
**HD**		7	21	60	83
**New transplant**		<5			
**PD**		<5		12	17
**Same transplant**		8	24		
**Sex**					
**Male**		16	48	41	57
**Female**		17	52	31	43
**Ethnicity**					
**Missing**		<5		0	
**Asian**		<5		5	7
**Black**		<5		8	11
**Mixed/Other**		<5		5	7
**White**		28	85	54	75
**IMD quintile**					
**Missing**		<5		0	
**1 (most deprived)**		7	21	23	32
**2**		7	21	13	18
**3**		7	21	17	24
**4**		5	15	9	13
**5 (least deprived)**		<5		10	14
**PRD**					
**Diabetes**		<5		7	10
**Glomerulonephritis**		<5		20	28
**Hypertension**		<5		<5	
**Missing**		20	61	7	10
**Other**		6	18	10	14
**Polycystic**		<5		<5	
**Pyelonephritis**		<5		9	13
**Renovascular**		<5		<5	
**Uncertain**		5	11	12	17
**Centre type**					
**Transplanting**		26	79	49	68
**Non-Transplanting**		7	21	23	32
**Pre-dialysis eGFR**	**Median**	10.3			
	**Lower quartile**	6.9			
	**Upper quartile**	14.4			

Table footnotes:
1: Includes 13 censored before 1 year and 20 missing covariate data.
2: Includes 7 missing covariate data and 65 with no eGFR data (patients on dialysis at year 1 only).
IMD: Index of Multiple Deprivation, PRD: Primary renal disease.
