# Supplementary material for: Inequalities in outcomes after kidney transplant failure in the UK
Source: PLoS One. 2026 Aug 26;21(8):e0356769. doi: 10.1371/journal.pone.0356769 (PMC13514003; doi:10.1371/journal.pone.0356769)
Supplement: Supplementary file 4 [file pone.0356769.s004.docx]

7^th^ June 2026

**Response to Academic Editor and Reviewers**

**Manuscript Number: PONE-D-26-08491**

Dear Dr Iseri,

Thank you for the comments on our manuscript and opportunity to submit a revised version. We detail below our responses and changes below.

***Journal Requirements:***

***1. Please ensure that your manuscript meets PLOS ONE's style requirements, including those for file naming. The PLOS ONE style templates can be found at*** 
[***https://journals.plos.org/plosone/s/file?id=wjVg/PLOSOne_formatting_sample_main_body.pdf***](https://journals.plos.org/plosone/s/file?id=wjVg/PLOSOne_formatting_sample_main_body.pdf) ***and*** 
[***https://journals.plos.org/plosone/s/file?id=ba62/PLOSOne_formatting_sample_title_authors_affiliations.pdf***](https://journals.plos.org/plosone/s/file?id=ba62/PLOSOne_formatting_sample_title_authors_affiliations.pdf)

Authors: The manuscript has been updated to comply with your formatting requirements.

***2. Thank you for stating the following in the Acknowledgments Section of your manuscript: 
“This work was funded by Kidney Research UK (Grant number: SG_MNRP_017_20231101). Samuel Westaway (ACF-2024-25-013) and Barny Hole (CL-2022-25-002) are funded by NIHR. The views expressed in this publication are those of the authors and not necessarily those of the NIHR, NHS or the UK Department of Health and Social Care.”
We note that you have provided funding information that is not currently declared in your Funding Statement. However, funding information should not appear in the Acknowledgments section or other areas of your manuscript. We will only publish funding information present in the Funding Statement section of the online submission form. 
Please remove any funding-related text from the manuscript and let us know how you would like to update your Funding Statement. Currently, your Funding Statement reads as follows: 
“This work was funded by Kidney Research UK (***[***https://www.kidneyresearchuk.org/***](https://www.kidneyresearchuk.org/)***) Grant number: SG_MNRP_017_20231101. Samuel Westaway (ACF-2024-25-013) and Barny Hole (CL-2022-25-002) are funded by NIHR (***[***https://www.nihr.ac.uk/***](https://www.nihr.ac.uk/)***). The views expressed in this publication are those of the authors and not necessarily those of the NIHR, NHS or the UK Department of Health and Social Care. The funding bodies did not play a role in the study design, data collection and analysis, decision to publish, or preparation of the manuscript.”
Please include your amended statements within your cover letter; we will change the online submission form on your behalf.***

Authors: The previously included ‘Funding Acknowledgements’ section has been removed from the manuscript. The Funding Statement has been amended to specify that Maria Pippias was the lead applicant for the Kidney Research UK grant. It has also been made clear that NIHR is the acronym for the UK’s National Institute for Health and Care Research.

*“This work was funded by Kidney Research UK (*[*https://www.kidneyresearchuk.org/*](https://www.kidneyresearchuk.org/)*); Maria Pippias was the lead applicant for this grant (Grant number: SG_MNRP_017_20231101). Samuel Westaway (ACF-2024-25-013) and Barny Hole (CL-2022-25-002) are funded by the UK’s National Institute for Health and Care Research (NIHR) (*[*https://www.nihr.ac.uk/*](https://www.nihr.ac.uk/)*). The views expressed in this publication are those of the authors and not necessarily those of the NIHR, NHS or the UK Department of Health and Social Care. The funding bodies did not play a role in the study design, data collection and analysis, decision to publish, or preparation of the manuscript.”*

***3. Your ethics statement should only appear in the Methods section of your manuscript. If your ethics statement is written in any section besides the Methods, please move it to the Methods section and delete it from any other section. Please ensure that your ethics statement is included in your manuscript, as the ethics statement entered into the online submission form will not be published alongside your manuscript.***

Authors: The section entitled ‘Ethical considerations and approval’ has been moved to the methods section as advised.

***4. Please include a separate caption for each figure in your manuscript.***

Authors: A separate caption has now been included for each figure, included after the paragraph in which they appear.

***Reviewer Comments:***

***Reviewer #1: Thank you for submitting this important and timely manuscript. As kidney transplants do not last forever. Management after kidney transplant failure is important but poorly understood. This paper attempts to sort out what happens to those whose kidney transplants fail. I have some questions about the methodology.***

1) ***how do the authors account for vintage of ESKD were they on dialysis for 5, years than had a transplant for 5 years is very different than a person who had a preemptive living donor transplant for 20 years.***

Authors: We agree that our cohort will represent a heterogenous group of patients. Unfortunately, missing data prevents us from including KRT duration as a covariate. We acknowledge this as a limitation which we have added to the discussion.

*“Cumulative duration of kidney replacement therapy may influence outcomes following transplant failure. Because some people began kidney replacement therapy for their native kidney failure outside of the UK, or prior to a centre joining the renal registry, we lack sufficient data completeness to adjust for this.”*

***2) how do the authors adjust for time? Is the primary model what happens to the patients within a year of being diagnosed with kidney transplant failure. I am confused because based on the Sankey a significant portion who "survive" this first year are eventually retransplanted (9.4%).***

Authors: It is correct that some patients diagnosed with transplant failure will go on to be re-transplanted after more than one year. We have expanded our ‘Kidney treatment status’ section of the methods to highlight that some patients diagnosed with transplant failure may not experience a change in kidney replacement therapy modality within a year. This updated text is provided below:

*“As in native kidney failure, there is variation in the time to KRT modality change after a patient reaches kidney transplant failure as defined as eGFR<15ml/min/1.73m^2^. For example, some people will be highly symptomatic, have rapidly declining function and change KRT within a year of transplant failure; others may not undergo a change in KRT treatment for many years, and have stable function and few symptoms despite an eGFR <15ml/min/1.73m^2^.”*

***3) eGFR analysis. I am not sure how to interpret the eGFR analysis. In the US, we usually initiate based on symptom rather than by eGFR. Why would they initiate at a high eGFR at a non-transplant centre?***

Authors: We completely agree that the question as to why another KRT is initiated at a higher eGFR at non-transplanting centres requires further research to understand the reasons for this. We are unable to investigate this further in our retrospective analysis of registry data: the reason for KRT change/dialysis initiation is not recorded in the UK renal registry data and therefore prospective or longitudinal mixed-methods research is required to investigate this further. Our experience from clinical practice suggests that the reason for initiation of dialysis varies between and within centres. The trigger for the initiation of dialysis varies: whilst it can be based on symptoms of uraemia or salt and water overload, it can also be based on hyperkalaemia, acidaemia, and, for some clinicians, an eGFR cut off. Clinicians and centres may vary in how much they attempt to manage the symptoms of kidney failure before making a change in KRT modality.

***Minor comment
The discussion is very good but kind of long. It would help to focus on findings by 1) sex, 2) race/ethnic minority, 3) social deprivations.
Otherwise I very much enjoyed the paper.***

Authors: Thank you. Paragraphs related to diabetic kidney disease being associated with mortality risk and the use of PD in areas of socioeconomic deprivation have been removed. We have also removed a paragraph in the limitations section regarding lack of comorbidity data, moving a paragraph from the main discussion to cover this point. Lastly, the potential for misclassification of deprivation level has been removed as a limitation, given we had previously included a reference highlighting this was unlikely to be a significant problem (88% of neighbourhoods in the most deprived decile in 2019 were also in the most deprived decile in the equivalent 2015 data).

***Reviewer #2: The authors have conducted a large registry based retrospective cohort study looking at outcomes post-transplant failure in about 5447 adult kidney transplant recipients. The topic is clinically relevant and aims to address gaps in knowledge regarding post–allograft failure outcomes and healthcare inequities. However, several key methodological limitations significantly affect the interpretation of the findings, particularly the conclusions regarding disparities in re-transplantation, and should be addressed prior to consideration for publication.

1) Socioeconomic status and ethnicity interaction: While both ethnicity and socioeconomic deprivation are examined, the manuscript does not explore their interaction which is a key limitation. There have been prior studies examining the role of SES and a recent study showed that a lower SES was associated with poorer graft and patient survival and the disparities persisted across ethnic groups by Michael Corr et al. in Transplantation - The Association of Socioeconomic Status on Kidney Transplant Access and Outcomes: Cohort Studies of England and Northern Ireland. Transplantation 110(4):p e866-e875, April 2026. | DOI: 10.1097/TP.0000000000005596.

Without assessing interaction or mediation, it is difficult to determine whether the observed ethnic disparities are independent or reflect underlying socioeconomic differences.***

Authors: We agree that it is important to try to understand the independent and dependent effects of ethnicity on outcomes after transplant failure.

The second author of our paper was a co-author of the paper you highlighted by Corr M et al. Transplantation 2026; 110(4):e866-75. In that paper the team formally tested for an interaction between SES and ethnicity, investigating whether the effect of SES on graft and patient survival differed by ethnicity. Interaction terms between deprivation quintile and ethnicity were not statistically significant (likelihood ratio test p=0.10 for graft survival; p=0.08 for patient survival). The analyses presented by Corr M et al. indicated that while both deprivation and ethnicity were independently associated with outcomes, there was no strong evidence that their effects differed across groups. This analysis was using a UK renal registry dataset as in our study. Similarly, Udayaraj et al found no evidence of an interaction between ethnicity and SES in a prior UK renal registry analysis exploring social deprivation, ethnicity and uptake of LKD transplantation (Udayaraj et al. Transplantation 2012; 93(6):p610-616).

We have expanded the following section in the discussion, and included reference to the paper by Corr M et al. and Udayaraj et al.:

*“Our finding of a reduced likelihood of pre-emptive first transplantation with higher socioeconomic deprivation is consistent with previous international research in first kidney transplants after native kidney failure (26-28) and re-transplantation after transplant failure.”*

Our manuscript represents initial work using routinely collected registry data to look for inequalities that might warrant further investigation in future mixed-methods work. A formal mediation analysis is not possible with registry data: a prospective cohort study or longitudinal study is required. Within the limitations of our study design, we have attempted to explore and address your important question of whether ethnic disparities “are independent of socioeconomic differences”: we adjusted for socioeconomic deprivation in our regression modelling when investigating the association between ethnicity and outcomes, recognising that it may be a partial or complete mediator. Even after adjustment for socioeconomic deprivation, we found persistent evidence of ethnic disparities suggesting in this limited analysis a direct effect, although this of course cannot be proven or quantified within this analysis.

***2) Short follow up period of one year: The restriction of primary analysis to one-year outcomes is an important limitation as key outcomes such as re-transplantation, death often occur beyond the first year, and this short follow-up may underestimate event rates and introduce bias when comparing subgroups with differing access to care pathways. Given that longer follow-up data (up to five years) are available, additional analyses examining outcomes over extended time periods or using a time-to-event approach may provide a more comprehensive assessment and strengthen the conclusions.***

Authors: We have presented outcomes up to 5 years post-transplant failure in our Sankey chart in Figure 1. As indicated in the Sankey, at 1 year post transplant failure, the vast majority (73.1%) of patients had received an alternative form of KRT, or died. We focussed on early outcomes at 1 year in this initial output: the patients included in this study are already under the care of a renal team and therefore one might expect earlier preparation for KRT after transplant failure and therefore early outcomes were of particular interest to our group.

We are planning further work to investigate further access to re-transplantation: this requires linkage of the UK renal registry dataset with NHS Blood and Transplant’s UK Transplant registry data to provide important information including cRF (PRA) and matchability score. In these later planned analyses we will be able to consider time to event analyses.  ***3) Absence of transplant specific parameters: Important data such as sensitization status and cPRA post allograft failure, prior transplant characteristics limits the ability to interpret the findings, this may become particularly significant across ethnic groups as immunologic matching and allocation factors may play a significant role. It would be important to either include these or address these as a key limitation for the study.***

Authors: This is certainly a limitation of our analysis. Unfortunately, data on PRA/calculated reaction frequency are not available in the UK renal registry, with this data held by NHS Blood and Transplant’s UK Transplant Registry. We are planning further work to investigate access to re-transplantation with a linked UK renal registry and UK Transplant registry dataset. We had detailed this in our discussion, but have now moved the relevant extract to be the lead paragraph of the limitations section to emphasise it:

*“Conclusions about the reasons for differences in access to re-transplantation are limited without data on tissue type, immunological sensitisation, re-listing and more detailed comorbidity metrics aside from primary renal disease. We plan to undertake a future analysis of linked UKRR and UK Transplant registry data to explore re-listing and re-transplantation patterns in the failed transplant population, alongside matchability scores, sensitisation metrics and comorbidities. Time to waitlisting after transplant failure, time to re-transplantation once listed and likelihood of living versus deceased kidney transplantation will be assessed. We hope this future work will increase our understanding of our observed ethnic, socioeconomic and sex-related inequalities.”*

***Minor comment: Definition of transplant failure: Defined as eGFR <15, this may be potentially misleading as some may have relatively stable eGFR without decline and may not require further intervention in that short period, it may be helpful to include the trajectory if possible***

Authors: There are no national or international consensus definitions of ‘kidney transplant failure’ which we recognise as problematic. We chose an eGFR value of <15ml/min/1.73m2 to represent transplant ‘failure’, since this value defines kidney failure in native kidney disease. We have highlighted this in our limitations section as below:

*“… we classified individuals with eGFR <15 ml/min/1.73m² as having transplant failure, although some may have had stable but poor kidney function without evidence of decline. As a result, our cohort includes some individuals with no clinical need to transition to another KRT modality, likely leading to an overestimation of the proportion alive with a “failed” transplant.”*

We have also expanded our ‘Kidney treatment status’ section of the methods to outline in more detail the fact that some patients diagnosed with transplant failure may not require a change in kidney replacement therapy modality within a year. This updated extract is below:

*“As in native kidney failure, there is variation in the time to KRT modality change after a patient reaches kidney transplant failure as defined as eGFR<15ml/min/1.73m2. For example, some people will be highly symptomatic, have rapidly declining function and change KRT modality within a year of transplant failure; others may not undergo a change in KRT treatment for many years, have stable function and few symptoms despite an eGFR <15ml/min/1.73m2.”*

Many thanks once again for the opportunity to submit a revised manuscript. We remain happy to receive any further comments to this response.

Warm regards,

Dr Samuel Westaway, on behalf of the authors

NIHR Academic Clinical Fellow in Renal Medicine

Population Health Sciences, Bristol Medical School

University of Bristol, United Kingdom
